# Supplementary material for: ATR kinase inhibition induces unscheduled origin firing through a Cdc7-dependent association between GINS and And-1
Source: Nat Commun. 2017 Nov 9;8:1392. doi: 10.1038/s41467-017-01401-x (PMC5680267; doi:10.1038/s41467-017-01401-x)
Supplement: Supplementary file 1 — Supplementary Information [file 41467_2017_1401_MOESM1_ESM.pdf]

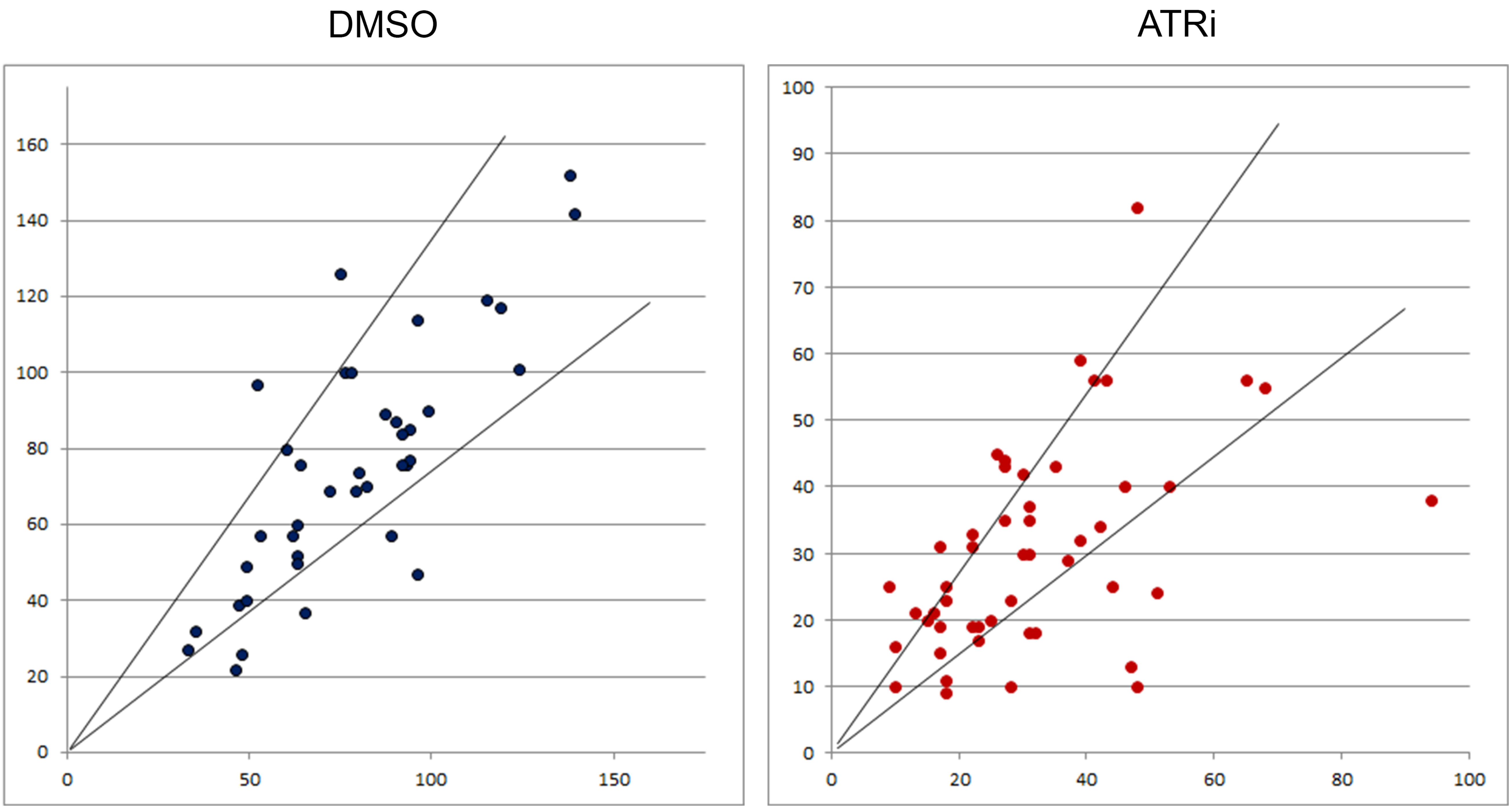

Supplementary Fig 1. Replication fork symmetry. Velocities of forks originating from the same origin but moving in the opposite directions are plotted against each other. The degree of asymmetry, as measured by absolute difference in velocity between the two forks, does not reach statistical significance (Wilcoxon p-value = 0.41).

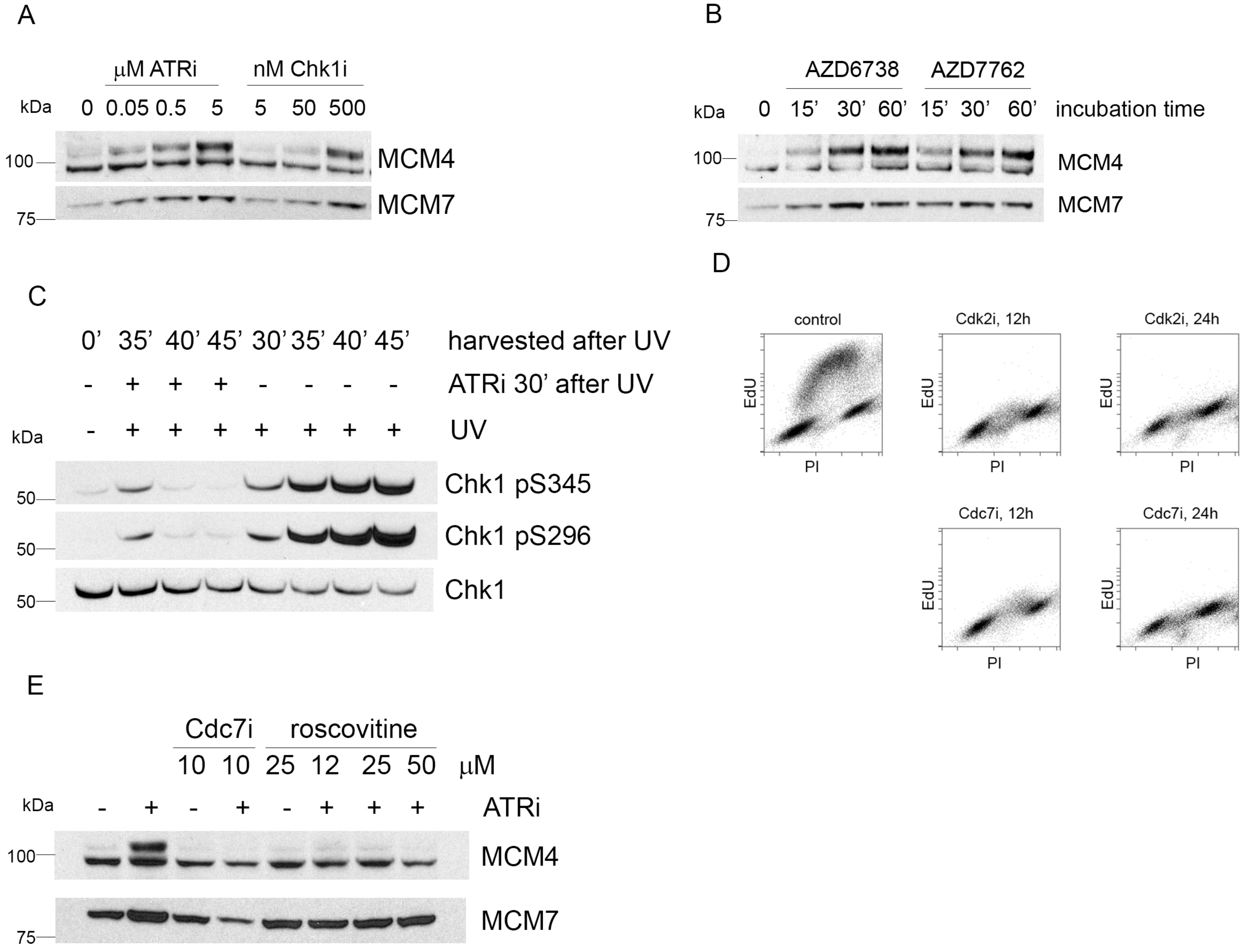

Supplementary Fig 2. 293T cells were fractionated as described in methods. Western blotting of the nuclease-insoluble chromatin fraction is shown. A. Cells were treated with various concentrations of AZD6738 or AZD7762 for 1h. B. Cells were treated with 5μM AZD6738 or 500nM AZD7762 for the indicated periods of time. C. 5uM of AZD6738 or vehicle was added to 293T cells 30 min after 10J m-1 UV irradiation. Cells were harvested at indicated time points after UV. Western blots of total cell lysates are shown. D. U2OS cells were treated with vehicle, 10μM of Cdc7 inhibitor or 10mM of Cdk2 inhibitor for indicated times. 10μM EdU was added to the cells for the last 30 minutes of incubation, cells were fixed and EdU was labeled with Alexa488-azide using the click reaction. After PI staining, cells were analyzed by flow cytometry to quantify EdU incorporation. E. 293T cells were treated with the indicated concentrations of Cdc7 inhibitor and roscovitine for 15 minutes before the addition of ATR inhibitor AZD6738. Cells were harvested and fractionated 1h later. Western blot analysis of the nuclease insoluble fraction is shown.

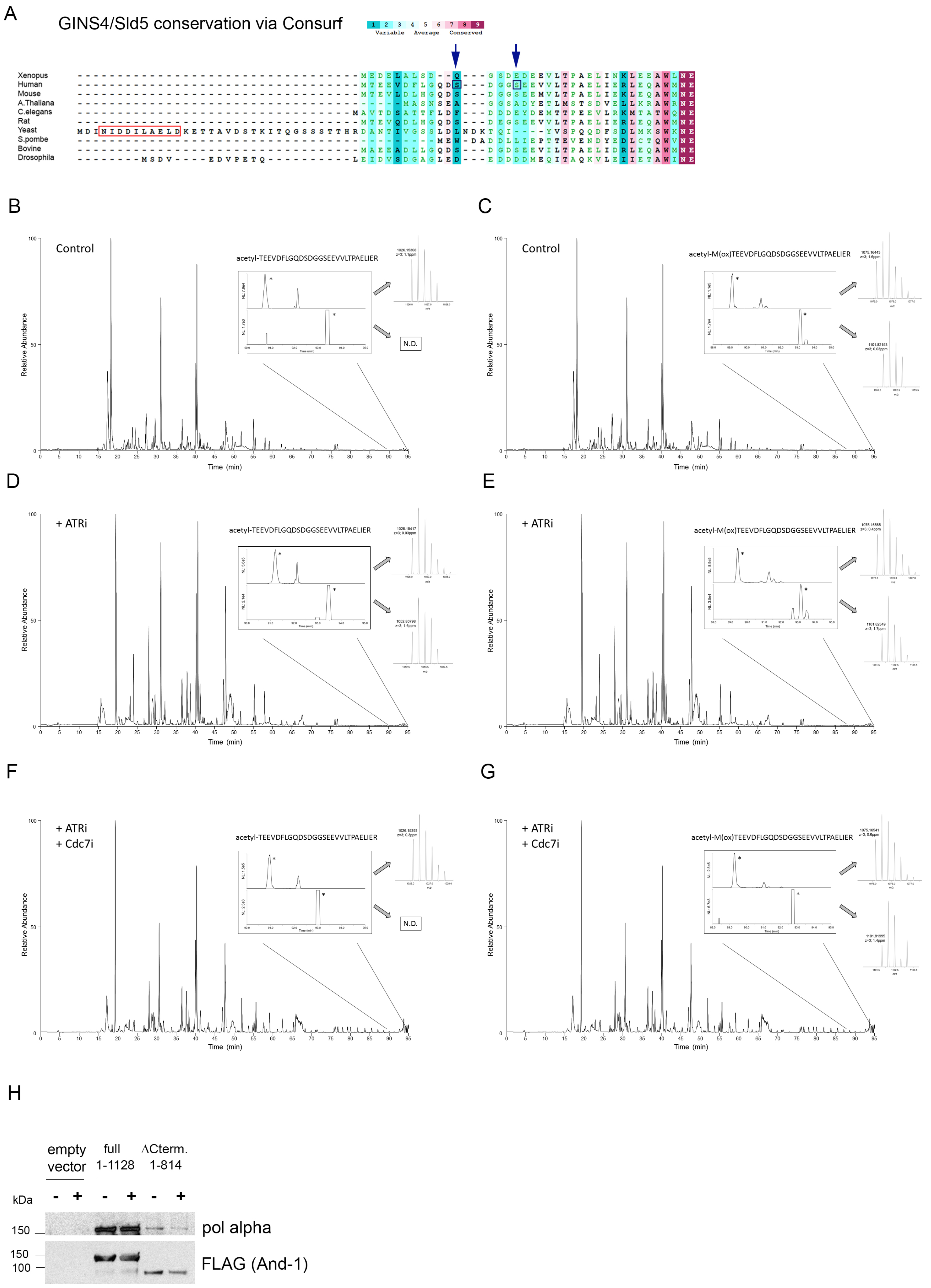

Supplementary Fig 3. A. Conservation analysis of GINS4/Sld5 subunit from various species was performed using Consurf server (http://bental.tau.ac.il/new_ConSurfDB/). N-terminal fragment is shown. Ctf4 binding peptide in yeast protein is marked with red frame, predicted Cdc7 phosphorylation sites on human GINS4 are marked with blue arrows. B-G. Base peak mass chromatograms from high resolution liquid chromatography-tandem mass spectrometry analyses of peptide digests generated from GINS4 immunoprecipitates from cells treated with vehicle (B and E), ATRi AZD6738 (C and F), and ATRi + Cdc-7i (D and H). Shown in the insets are mass chromatograms representing the unphosphorylated (inset, top panel) and phosphorylated (inset, bottom panel) forms of methionine (oxidized)-containing (B-D) and methionine-cleaved (E-G) N-terminal peptide isoforms (retention time for each peptide isoform in the reversed phase liquid chromatography gradient is denoted by an asterisk). Isotopic envelopes for each peptide molecular ion observed are shown to the right of each inset with the observed m/z, charge state (z), and mass measurement accuracy (ppm, parts per million) for the unphosphorylated (upper mass spectrum) and phosphorylated (lower mass spectrum) N-terminal peptide isoforms (N.D., not detected). H. 293T cells were trans fected with empty vector or vector expressing And-1-FLAG. 48h later cells were treated with 5μM ATRi (AZD6738) for 1h. Cells were lysed, And-1-FLAG was immunoprecipitated and eluted with FLAG peptide. Western blot analysis of the elution samples is shown.

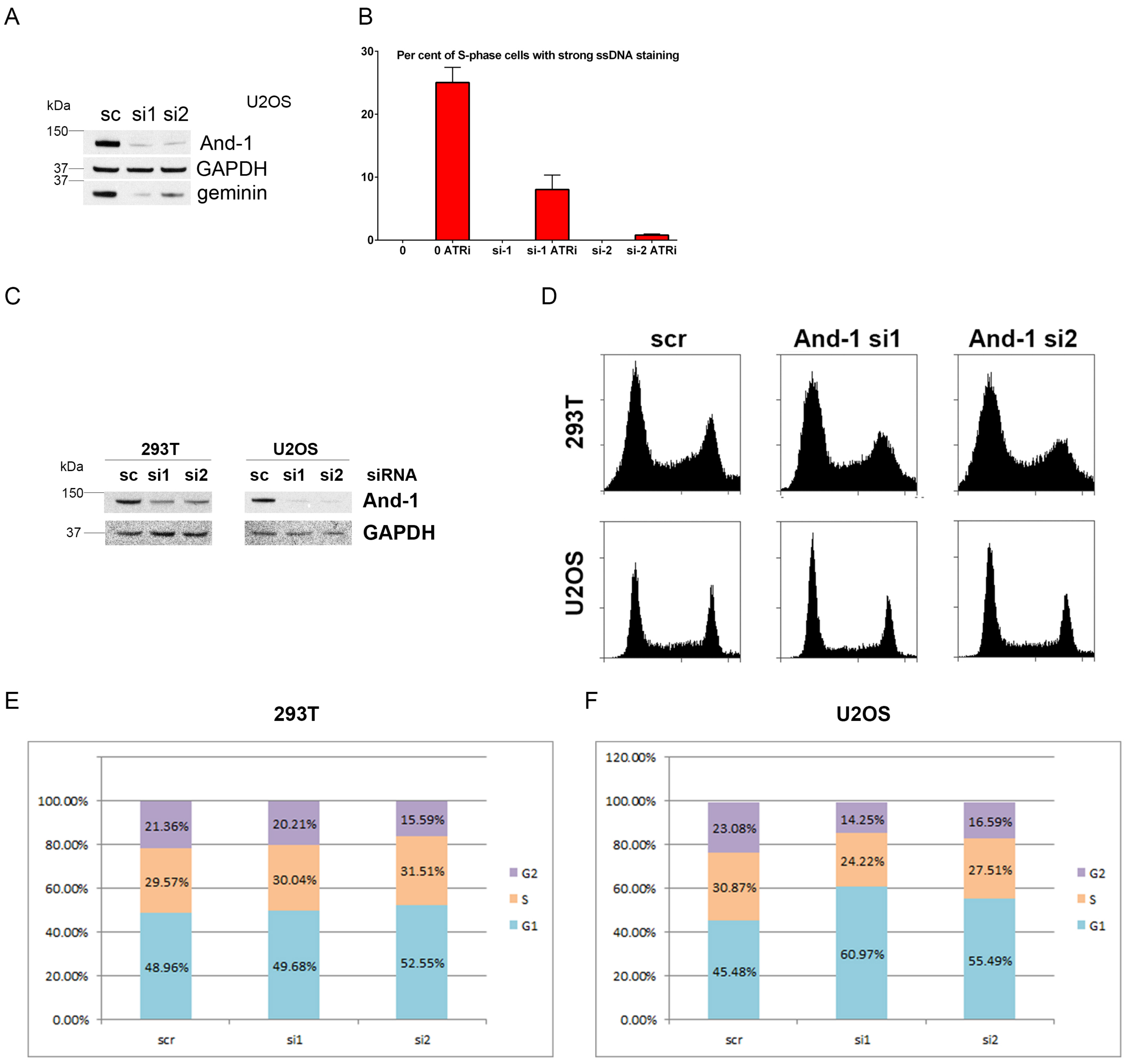

Supplementary Fig 4. A. Whole cell lysate western blot of U2OS cells transfected with siRNAs against And-1. B. U2OS cells were transfected with siRNAs against And-1. ATRi-induced ssDNA was detected as in Fig.5A 48h after siRNA transfection. C. Whole cell lysate western blot of 293T or U2OS cells transfected with siRNAs against And-1. Samples correspond to the experiment shown in panels D-F. D-F. Cell cycle analysis of 293T or U2OS cells 48h after the transfection with siRNAs against And-1

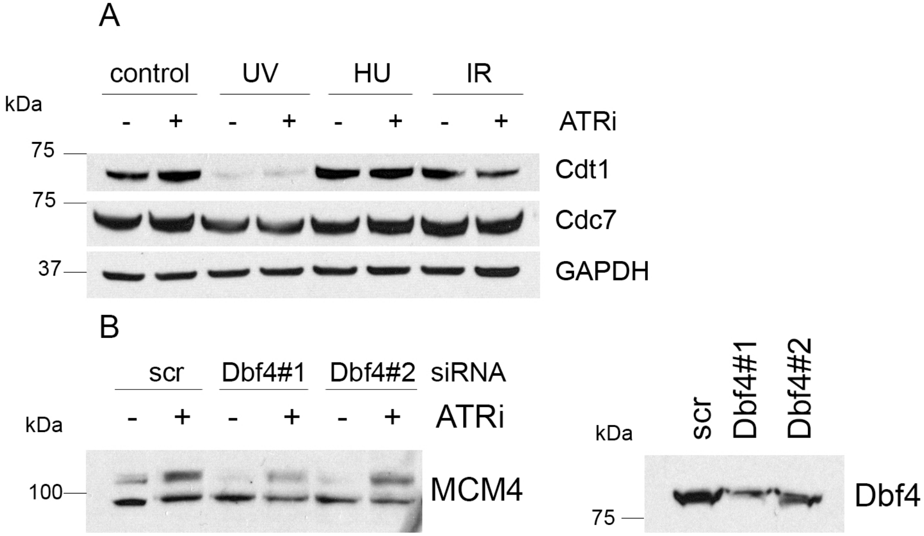

Supplementary Fig 5. A. 293 cells were treated with vehicle or 5µM AZD6738 for 15 minutes before DNA damage (10J m-1 UV, 5mM HU or 5 Gy IR). Cells were harvested 1 h after DNA damage, western blot analysis of the total cell lysates is shown. B. 293T cells were transfected with siRNAs against Dbf4. 48h after transfection cells were treated with vehicle or 5µM AZD6738 for 1h, harvested and fractionated. Western blot of MCM4 in the nuclease-insoluble fraction is shown. Western blot of total cell lysates is shown as a control of the knockdown efficiency

1f, PSF3

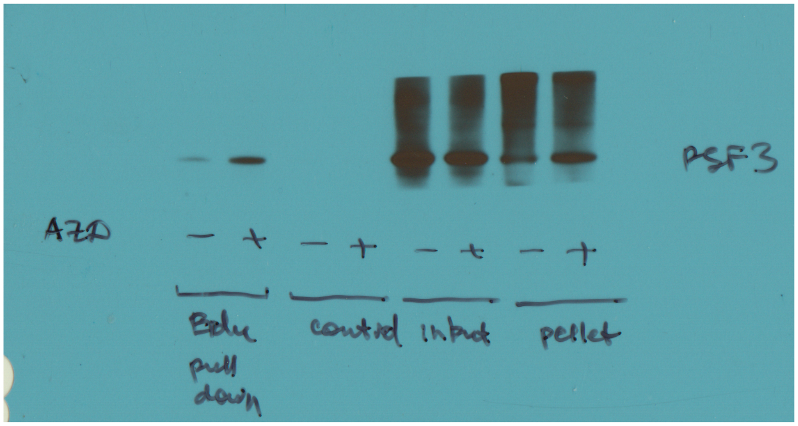

1f, H3

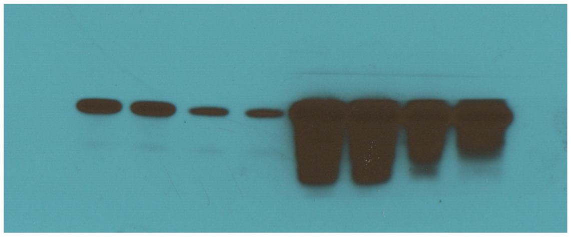

2b, MCM4

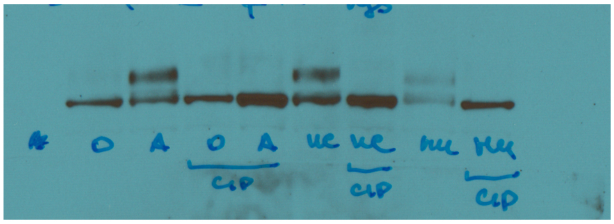

3d, PSF3

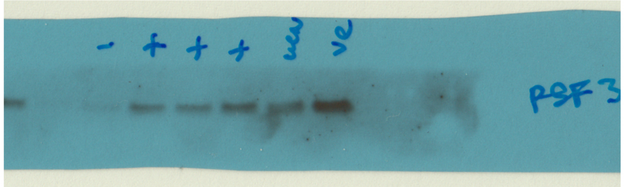

3d, FLAG

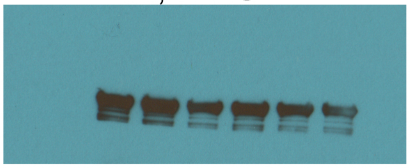

3e, PSF3

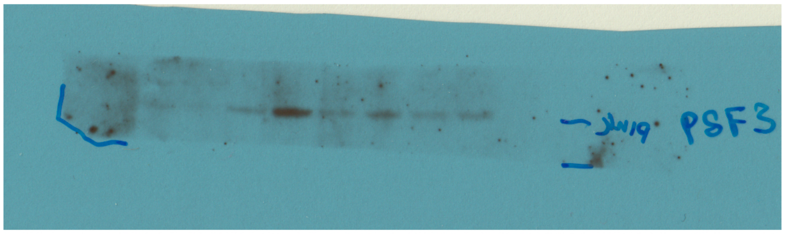

3e, FLAG

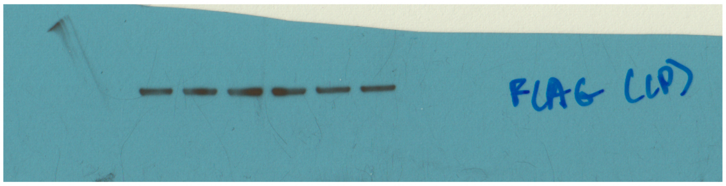

3e, pol alpha

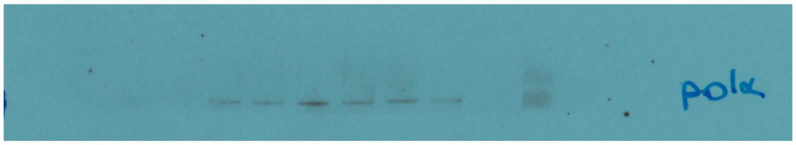

3f, GST

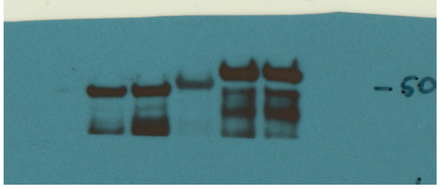

3f, And-1

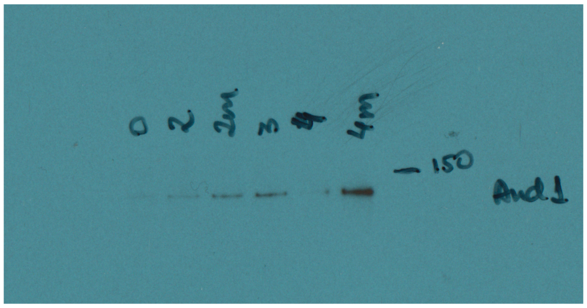

Supplementary Fig. 6. Uncropped versions of the important western blots
