# Supplementary material for: ATR kinase inhibition induces unscheduled origin firing through a Cdc7-dependent association between GINS and And-1
Source: Nat Commun. 2017 Nov 9;8:1392. doi: 10.1038/s41467-017-01401-x (PMC5680267; doi:10.1038/s41467-017-01401-x)
Supplement: Supplementary file 3 — Description of Additional Supplementary Files [file 41467_2017_1401_MOESM3_ESM.pdf]

**Description of Additional Supplementary Files**

File Name: Supplementary Data 1
Description: The nuclease-insoluble chromatin proteome in 293T cells treated with vehicle or ATRi AZD6738 identified using high resolution liquid chromatography-tandem mass spectrometry (LC-MS/MS). Protein abundance differences were determined by spectral counting (PSM, peptide spectral match).
